# Prevalence of developmental dental hard-tissue anomalies and association with caries and oral hygiene status of children in Southwestern, Nigeria

**DOI:** 10.1186/s12903-016-0236-6

**Published:** 2016-07-07

**Authors:** Bamidele O. Popoola, Nneka Onyejaka, Morenike O. Folayan

**Affiliations:** Department of Child Oral Health, College of Medicine, University of Ibadan, Oyo State, Nigeria; Department of Child Dental Health, Obafemi Awolowo University Teaching Hospitals Complex, Ile-Ife, Nigeria; Department of Child Dental Health, Obafemi Awolowo University, Ile-Ife, Nigeria; Department of Child Oral Health, Faculty of Dentistry, College of Medicine, University of Ibadan, Oyo State, Nigeria

**Keywords:** Developmental, Dental hard-tissue, Anomalies, Dental caries, Oral hygiene

## Abstract

**Background:**

Developmental dental hard tissue anomalies are often associated with oral health problems. This study determined the clinical prevalence of developmental dental hard tissue anomalies in the permanent dentition of children resident in southwestern Nigeria and its association with dental caries and poor oral hygiene status.

**Methods:**

This was a cross-sectional study recruiting 1565 school children, 12 to 15 year old attending schools in Ibadan, Oyo State and Ile-Ife, Osun State. All eligible study participants had oral examinations conducted to determine presence of developmental hard dental tissue anomalies, caries and oral hygiene status. The prevalence of developmental dental hard tissue anomalies was determined. Logistic Poisson regression was used to determine the association of between developmental dental hard tissue anomalies, caries and oral hygiene status.

**Results:**

Only 65 (4.2 %) children had clinically diagnosed developmental dental hard tissue anomalies. The most prevalent anomaly was enamel hypoplasia (2.2 %). More females (*p* = 0.003) and more children with middle socioeconomic class (*p* = 0.001) had enamel hypoplasia. The probability of having poor oral hygiene was significantly increased for children with developmental dental anomalies (APR: 0.07; 95 % CI: 0.03 – 0.12; *p* = 0.002). The probability of having caries was insignificantly increased for children with developmental dental hard tissue anomalies (APR: 0.005; 95 % CI: −0.03 – 0.04; *p* = 0.08).

**Conclusion:**

The most prevalence clinically detectable developmental dental hard tissue anomalies for the study population was enamel hypoplasia. The presence of developmental dental hard tissue anomalies significantly increased the chances of having poor oral hygiene but not caries. Further studies are required to understand if poor oral hygiene is associated with dental caries in children with developmental dental hard tissue anomalies.

## Background

Developmental dental hard tissue anomalies cover a range of anomalies that affect tooth number, shape, size and tooth structures. Its prevalence varies between countries and tribes. For example congenitally missing teeth is the most prevalent dental anomaly found in Indians [1, 2], Saudi Arabians [3], Turkish [4] and Norwegian children [5]. In Nigeria however, enamel hypoplasia is the most common dental anomaly in children while hypodontia is a rare clinical feature [6, 7].

A number of these anomalies can be diagnosed clinically and prompt management is important for a number of reasons: they are associated with oral health problems including malocclusion [8], caries [9] poor oral hygiene [10] and aesthetic concerns [11]. They could also predispose to functional problems and other diseases [1]. In addition, the developmental dental hard tissue anomalies may be associated with syndromes, especially when the anomalies are multiple [12, 13].

Past studies have demonstrated an association between developmental dental hard tissue anomalies and poor oral health status: developmental enamel defects are associated with increased risk for caries [14–16]; and supernumerary teeth can also lead to gingivitis and caries due to plaque retention in inaccessible areas [17]. Hypodontia and hyperdontia are both associated with malocclusion [18] and malocclusion increases the risk for caries [19] and poor oral hygiene [20]. These associated oral health disorders could impact negatively on the quality of lives of affected persons [7, 21].

However, little is known about how developmental dental hard tissue anomalies constitute a risk factor for dental caries and poor oral hygiene in the study population where the use of fluoride containing dentifrices is high [22], caries prevalence and severity is low [23] and the proportion of children with poor oral hygiene is low [24]. This study therefore determined the association of clinical presence of developmental dental hard tissue anomalies, caries and poor oral hygiene. This study also builds on a prior report that determined the clinical prevalence of developmental dental hard tissue anomalies in the mixed dentition [7]: this study determined the clinical prevalence of developmental dental hard tissue anomalies in the permanent dentition in Southwestern Nigeria.

## Methods

### Study setting

This was a cross-sectional study conducted in secondary schools in two of the six states in South-western Nigeria. The States are Osun and Oyo States. The states were selected based on the ease of access of the study team to school pupils (the institutions of the investigators are located within the states). To ensure access to children with diverse socioeconomic background, Ibadan (an urban region in Oyo State) and Ile-Ife (a suburban region in Osun State) were considered appropriate study sites.

### Sampling procedure

In each state, pupils from secondary schools in the five local government areas that make up Ibadan metropolis and one of the four local government areas that make up Ile-Ife were recruited using a multi-prong approach.

The sample was first proportionally distributed amongst the Local Government Areas and then proportionately distributed between the private and public schools in the Local Government Areas. Each public and private secondary school from which participants emerged were selected from the sampling frame based on a constant of K = 10 (The constant K was chosen to be 10 based on the least number of schools in a local government such that every 10th school in each local government was selected for the study). For this study, proportionate representation of private and public schools was important so as to ensure representation of children from all socio-economic strata in the study sample. Usually children from the high socioeconomic strata attend private schools and those from the low socioeconomic strata attend public schools.

In each school, the classes with the highest number of pupils who met the age eligibility criteria were selected for the study. All children within the age range for the study were recruited into the study. Recruitment in each school continued till the sample size for the school was reached.

### Study population

The study population included all children who met the following criteria: 12 years to 15 years of age, parental consent for study participation was obtained, student assent for study participation was obtained. The age 12 years was selected as the minimum age since mean age of eruption of the second permanent molar in Nigerian children is approximately 12 years [25].

### Sample size

The sample size for the study was estimated by use of the Leslie Fischer’s formula [26] for study populations of more than 10,000 at a 95 % confidence level with 26.6 % prevalence of developmental dental hard tissue anomalies [7]. It was determined that it would be necessary to examine a minimum of 303 children per State for the study to be powered enough to determine the prevalence of developmental dental hard tissue anomalies in the region.

### Data collection

Data was collected through personal interviews of study participants and conduct of oral examinations. Data was collected using the same structured questionnaire administered by Temilola et al. [7] in their study. The questionnaire elicited information on the child’s socio-demographic characteristics (age, sex, and socioeconomic status). Socioeconomic status for the purpose of this study was obtained through a multiple item-scoring index combining the mother’s level of education with the occupation of the father; each child was allocated to a social stratum I to V, with social stratum V being the lowest. The social classification system has been well-tested and found valid and reliable for the Nigerian environment [27, 28]. Each child was classified as being in high socioeconomic class (strata 1 and II), middle socioeconomic class (strata III), and low socioeconomic class (strata IV and V).

All children eligible to participate in the study had an oral examination. A paediatric dentist conducted the clinical examination for study participants in Oyo and Osun States respectively. These dentists were conversant with normal and pathological dental features and both had more than 10 years of clinical practice experience. The children were examined under natural light while sitting on a chair. The teeth were examined wet and debris was removed with a piece of gauze when present.

### Developmental dental hard tissue anomalies

All developmental dental hard tissue anomalies that were clinically observable were recorded. The diagnostic criteria for developmental dental hard tissue anomalies adopted by Temilola et al. [7] were used for this study. The developmental dental hard tissue anomalies diagnosed were talon cusp, microdontia, macrodontia, gemination, fusion, enamel hypoplasia, dens evaginatus, dens invaginatus, supernumerary, hypodontia, tooth transposition and notched incisor.

### Caries assessment

The numbers of decayed, filled and missing teeth (DMFT) were noted for children with caries. The DMFT was determined based on the WHO Oral Health Survey methods [29]. The examination for dental caries was conducted with a sterile mouth mirror and dental caries explorer using natural light outdoors with the child seated on a chair. The examination of the teeth was done in an orderly manner from one tooth to another. Examination for dental caries included all surfaces.

To arrive at a DMFT score for an individual child, three values were determined: the number of teeth with carious lesions, the number of extracted teeth due to caries, and the number of teeth with fillings or crowns [30]. Where a missing tooth was identified, efforts was made to explore the reason for the lost tooth from the child and where possible, from the parent through a phone call placed through to seek clarification on reason for missing teeth. Only teeth extracted due to caries were recorded as missing. The number of carious, missing due to caries and filled teeth are summed together to give the DMFT score for each participant. For analysis purposes, caries was also classified as present or absent.

### Oral hygiene assessment

The oral hygiene status was determined using the oral hygiene index (OHI-S) by Greene and Vermillion [31]. The OHI-S comprises debris and calculus scores on selected tooth surfaces. The following were the index teeth: the first molars but sometimes the second were used if the first molars were missing. The buccal surfaces of upper molars and the lingual surfaces of the lower molars were examined. In the anterior portion of the mouth, the labial surfaces of the upper right and the lower left central incisors were examined.

The debris and calculus scores were added and divided by the number of surfaces examined to give the OHI-S score as recommended. Oral hygiene was classified as good, fair, or poor when the score ranges were 0.0–1.2, 1.3–3.0, and >3.0, respectively.

### Standardization of examiners

The two examiners are trained Paedodontists, practicing for a minimum of 10 years. They both undertook a series of calibration exercises to ensure the validity of their evaluations. The exercises was based on the protocol by Temilola et al. [7] for dental anomaly, the WHO criteria for the diagnosis of caries [30] and the OHI-S index described by Green and Vermillion [31]. The examiners had several sessions reviewing clinical photographs and repeated practices on examination of lesions, using clinical photographs. This training was followed by examination of live patients.

### Data analysis

For ease of analysis, socioeconomic status in this study was regrouped into three levels: high (upper and upper middle classes), middle (middle class), and low (lower middle and lower classes). This categorization was used to test associations and for logistic regression analysis. This categorization of socioeconomic status was previously used by Folayan et al. [32]. Descriptive analysis was conducted to determine the prevalence of ECC in the study population for each age, each sex, and each socioeconomic stratum. The Pearson’s Chi-squared test or Fisher’s exact test was used to test associations between developmental dental hard tissue anomalies and (i) sex, (ii) age, (iii) socioeconomic status, (iv) presence of caries and (v) oral hygiene status. Multivariate logistic regression was also conducted using the Poisson regression analysis, to determine the association between presence of developmental dental hard tissue anomalies, caries and poor oral hygiene. The estimated coefficients, expressed as prevalence ratios (PRs), and their 95 % confidence intervals were also calculated. Statistical analysis was done with Intercooled STATA (release 12). Simple proportions were computed. Statistical significance was inferred at *p* < 0.05.

## Results

One thousand, five hundred and sixty five children were recruited into the study. No child eligible to participate in the study refused study participation. Age, sex, and socioeconomic class of study participants recruited for the study are as shown in Table 1. Sixty five (4.2 %) children had developmental dental hard tissue anomalies. Of the 65 children who had developmental dental hard tissue anomalies, none had two or more developmental dental hard tissue anomalies. There was no significant age (*p* = 0.46), sex (*p* = 0.44) and socio-economic (*p* = 0.48) difference in the proportion of children who had or did not have developmental dental hard tissue anomalies.

**Table 1:** Socio-demographic profile of respondents with and without dental hard tissue anomalies (*N* = 1565)

Variables	Dental hard tissue anomaly present (*n* = 65)	Dental hard tissue anomaly absent (*n* = 1500)	Total (*N* = 1565)	*P* value
Sex				
Male	24 (36.9 %)	627 (41.8 %)	651 (41.6)	0.44
Female	41 (63.1 %)	873 (58.2 %)	914 (58.4 %)	
Age				
12 years	25 (38.5 %)	456 (30.4 %)	481 (30.7 %)	0.46
13 years	18 (27.7 %)	498 (33.2 %)	516 (33.0 %)	
14 years	13 (20.0 %)	366 (24.4 %)	379 (24.2 %)	
15 years	9 (13.8 %)	180 (12.0 %)	189 (12.1 %)	
Socio-economic status				
Low	21 (32.3 %)	499 (33.3 %)	520 (33.2 %)	0.48
Middle	32 (49.2 %)	640 (42.7 %)	672 (43.0 %)	
High	12 (18.5 %)	361 (24.0 %)	373 (23.8 %)	
Caries status				
Present	8 (12.3 %)	165 (11.0 %)	173 (11.0 %)	0.70
Absent	57 (87.7 %)	1335 (89.0 %)	1392 (89.0 %)	
Oral hygiene status				
Good	10 (15.4 %)	362 (24.1 %)	372 (23.8 %)	0.03
Fair	44 (67.7 %)	1009 (67.3 %)	1053 (67.3 %)	
Poor	11 (16.9 %)	129 (8.6 %)	140 (8.9 %)	

There was also no significant difference in the proportion of children with or without developmental dental hard tissue anomalies who had caries (*p* = 0.70). However, significantly more children with developmental dental hard tissue anomalies had poor oral hygiene (*p* = 0.03) when compared with children who did not have developmental dental hard tissue anomalies.

Table 2 shows the prevalence of each dental anomaly and the distribution by gender and socioeconomic class. The most prevalent developmental dental hard tissue anomaly found in the study population was enamel hypoplasia (54.0 %). Other lesions identified were microdontia (40.0 %), fusion/gemination (1.5 %), dens evaginatus (1.5 %), dens invaginatus (1.5 %) and talons cusp (0.06 %). Significantly more females (63.1 %) than males (36.9 %) had enamel hypoplasia (*p* = 0.003). Also, significantly more children with middle (49.2 %) socioeconomic class had enamel hypoplasia when compared with children with low (32.3 %) and high (18.5 %) socioeconomic class (*p* = 0.001).

**Table 2:** Dental anomalies by sex and socio-economic status (*N* = 65)

Dental hard-tissue anomaly	Number of cases affecting Male *n* = 24	Number of cases affecting Female *n* = 41	Number of cases affecting Low SES *n* = 21	Number of cases affecting Middle SES *n* = 32	Number of cases affecting High SES *n* = 12	Prevalence of lesion *N* = 65
Enamel hypoplasia	11 (45.8 %)	24 (58.6 %)	11 (52.4 %)	15 (46.9 %)	9 (75.0 %)	35 (54.0 %)
Microdontia	12 (50.0 %)	14 (34.2 %)	9 (42.9 %)	14 (43.8 %)	3 (25.0 %)	26 (40.0 %)
Dens Evaginatus	0 (0.0 %)	1 (2.4 %)	0 (0.0 %)	1 (3.1 %)	0 (0.0 %)	1 (1.5 %)
Fusion/germination	0 (0.0 %)	1 (2.4 %)	0 (0.0 %)	1 (3.1 %)	0 (0.0 %)	1 (1.5 %)
Talon cusp	1 (4.2 %)	0 (0.0 %)	1 (4.7 %)	0 (0.0 %)	0 (0.0 %)	1 (1.5 %)
Dens invaginatus	0 (0.0 %)	1 (2.4 %)	0 (0.0 %)	1 (3.1 %)	0 (0.0 %)	1 (1.5 %)
Macrodontia	0 (0.0 %)	0 (0.0 %)	0 (0.0 %)	0 (0.0 %)	0 (0.0 %)	0 (0.0 %)
Supernumerary	0 (0.0 %)	0 (0.0 %)	0 (0.0 %)	0 (0.0 %)	0 (0.0 %)	0 (0.0 %)
Transposition	0 (0.0 %)	0 (0.0 %)	0 (0.0 %)	0 (0.0 %)	0 (0.0 %)	0 (0.0 %)
Notch incisor	0 (0.0 %)	0 (0.0 %)	0 (0.0 %)	0 (0.0 %)	0 (0.0 %)	0 (0.0 %)
Hypodontia	0 (0.0 %)	0 (0.0 %)	0 (0.0 %)	0 (0.0 %)	0 (0.0 %)	0 (0.0 %)
Total	24 (100.0)	41 (100.0)	21 (100.0)	32 (100.0)	12 (100.0)	65 (100.0)

Table 3 highlights the proportion of children with developmental dental hard tissue anomalies by their caries and oral hygiene status. The proportion of children who had enamel hypoplasia (*p* = 0.43) and microdontia (*p* = 0.53) who also had caries were not significantly more than those children who had the developmental dental hard anomalies and did not have caries. Likewise, the proportion of children with enamel hypoplasia who had good, fair and poor oral hygiene was not significantly different (*p* = 0.36). However, more children with microdontia had fair oral hygiene compared with children with microdontia who had good and poor oral hygiene (*p* = 0.01).

**Table 3:** Developmental dental hard tissue anomalies by caries and oral hygiene status (*N* = 65)

Dental hard-tissue anomaly	Number of cases with caries *n* = 8	Number of cases without caries *n* = 57	Number of cases with good oral hygiene *n* = 10	Number of cases with fair oral hygiene *n* = 44	Number of cases with poor oral hygiene *n* = 11	Prevalence of lesion (*N* = 65)
Enamel hypoplasia	2 (25.0 %)	33 (57.8 %)	5 (50.0 %)	26 (59.1 %)	4 (36.4 %)	35 (54.0 %)
Microdontia	4 (50.0 %)	22 (38.6 %)	4 (40.0 %)	15 (34.0 %)	7 (63.6 %)	26 (40.0 %)
Dens Evaginatus	1 (12.5 %)	0 (0.0 %)	0 (0.0 %)	1 (2.3 %)	0 (0.0 %)	1 (1.5 %)
Fusion/germination	0 (0.0 %)	1 (1.8 %)	0 (0.0 %)	1 (2.3 %)	0 (0.0 %)	1 (1.5 %)
Talon cusp	0 (0.0 %)	1(1.8 %)	1 (10.0 %)	0 (0.0 %)	0 (0.0 %)	1 (1.5 %)
Dens Invaginatus	1 (12.5 %)	0 (0.0 %)	0 (0.0 %)	1(2.3 %)	0 (0.0 %)	I (1.5 %)
Total	8 (100.0 %)	57 (100.0 %)	10 (100.0)	44 (100.0 %)	11 (100.0 %)	654 (100.0 %)

Table 4 highlights the odds of having caries and poor oral hygiene in children with developmental dental hard tissue anomalies adjusting for the effect of age, sex and socioeconomic class, caries and poor oral hygiene. Caries could increase the risk for poor oral hygiene [33] and poor oral hygiene could increase the risk for caries: [34, 35]. Age, sex and socioeconomic status are risk factors for caries [36–38] and poor oral hygiene [39, 40]. It was therefore important to adjust for these possible confounders in this regression model.

**Table 4:** Multivariate analysis of factors associated with presence of developmental dental hard tissue anomalies (*N* = 1565)

Variables	Adjusted Prevalence Ratio (APR)	Std. Err.	*P* value	95 % Conf. Interval
Oral hygiene status				
Good oral hygiene status	1.00	-	-	-
Fair oral hygiene status	0.02	0.02	0.14	−0.007 - 0.05
Poor oral hygiene status	0.07	0.03	0.002	0.03 – 0.12
Caries status				
Absence of caries	1.00	-	-	-
Presence of caries	0.005	0.02	0.77	−0.03 – 0.04
Gender				
Male	1.00	-	-	-
Female	−0.006	0.01	0.64	−0.03 – 0.02
Socioeconomic status				
High socioeconomic class	1.00	-	-	-
Middle socioeconomic class	−0.001	0.02	0.95	−0.03 – 0.03
Low socioeconomic class	−0.007	0.02	0.68	−0.04 – 0.03

Children with developmental dental hard tissue anomalies had significantly increased probability of having poor oral hygiene when compared with children who did not have developmental dental hard tissue anomalies (APR: 0.07; 95 % CI: 0.03 – 0.12; *p* = 0.002). Children with developmental dental hard tissue anomalies had insignificantly increased probability of having caries when compared with children who did not have developmental dental hard tissue anomalies (APR: 0.005; 95 % CI: −0.03 – 0.04; *p* = 0.077).

## Discussion

This study contributes to body of literature on the prevalence of developmental dental anomalies in the permanent dentition. The study found that the prevalence of developmental dental hard tissue anomalies in the study populations was low, there was no sex, age or socio-economic differences in the prevalence of developmental dental hard tissue anomalies in the permanent dentition of children and there was no significant association between the presence of developmental dental hard tissue anomalies and dental caries. There was however, a significant association between the presence of developmental dental hard tissue anomalies and oral hygiene status; more children with developmental dental hard tissue anomalies were likely to have poor oral hygiene. Also, more female children and more children with middle socioeconomic status were likely to have enamel hypoplasia.

The study has a few limitations. First, while we studied 11 different developmental dental hard tissue anomalies, only six lesions (enamel hypoplasia, microdontia, dens evaginatus, fusion/germination, Talon cusp and dens invaginatus) were identified in the study population. Of these 93.8 % of the lesion were enamel hypoplasia and microdontia. The study findings may therefore bear more relevance to these two lesions than the entire range of developmental dental hard tissue anomalies. Second, the diagnosis of microdontia and macrodontia was based on visual examination and not by measuring the dimensions of the teeth using casts thus increasing the potential to introduce bias. Third, the assessment of caries was based on the WHO criteria. This implies that the number of teeth with caries detected in this study would be lower than the true prevalence since early enamel caries would not be detected. Fourth, though the field workers were experienced dentists who had practiced for many years in the field of paediatric dentistry, not conducting intra- and inter-examiner reliability increases the risk for disparity in diagnosis of lesion. We however feel that the disparity cannot be large enough to cause significant differences in findings of the examiners on the field since the examiners were all trained in the same institution by the same senior consultant, and had conducted multiple oral health surveys as a team in the past.

Despite these limitations, this study provides further evidence about risk factors for caries in the study environment. Prior studies have shown that the caries prevalence in the study population was low [32, 41, 42] and even lower than the caries prevalence in many other developing and developed countries. The risk and protective factors for caries in the study environment are also not well understood [32]. This study provides evidence that the presence of developmental dental hard tissue anomalies does not increase the probability of children having caries in the study population.

Of importance is the significant association between developmental dental hard tissue anomalies and poor oral hygiene. The presence of dental hard tissue anomalies increases difficulty in tooth cleaning [22]. It also increases malocclusion, which also increases the risk for plaque retention and poor oral hygiene [42, 43]. The finding of this study is therefore consistent with prior observations [44, 45] and has programmatic implications for managing adolescents. Adolescents with developmental dental hard tissue anomaly should be treated as having high risk for poor oral hygiene and should therefore be recalled more frequently for dental visits with particular emphasis on educating them about oral toileting including possible use of adjunctive therapies. This is important as oral health affect adolescents perception of body image, self-esteem and mental health [46, 47].

This study found a non-significant association between caries and presence of enamel hypoplasia unlike the findings of some previous studies [48–51]. While Vargas-Ferreira et al’s [51] meta-analysis strongly indicates that developmental defects of the enamel such as enamel hypoplasia is a risk factor for caries, this study finding indicates that enamel hypoplasia is not a risk factor for caries in the study population from a sub-urban developing country where the caries prevalence and severity is low [52]. However, the non-significant association between developmental dental hard tissue anomalies and caries and the significant association between developmental dental hard tissue anomalies and poor oral hygiene may highlight the probable pathophysiology of caries associated with developmental dental hard tissue anomalies: caries results as a secondary outcome of poor oral hygiene and not through a direct pathway. This postulation would need further studies, as there are multiple inter-related factors that may increase the susceptibility of teeth with developmental dental hard tissue anomalies to caries.

The study finding on gender and socioeconomic class differences in the prevalence of enamel hypoplasia differed from the findings of Robles et al. [53] in Spain who showed increased prevalence increased prevalence of developmental defects of the enamel (inclusive of enamel hypoplasia) in males and in children from middle and low socioeconomic status. The increasing risk for developmental defects of the enamel with decreasing socioeconomic status had been established, with this association linked to poor nutritional status [54]. However, the differences in the prevalence of developmental defects of the enamel by gender remains unclear with authors identifying male at greater risks [55, 56], some identifying females at increased risk [57, 58] while others show no gender association [59, 60]. Many of these studies assessed enamel defects, regardless of whether it was opacity or hypoplasia.

This study was a school based study implying that children in Southwestern Nigeria who do not attend school have been left out of this survey as reports show that a high proportion of children in Nigeria are out of school [61]. This limits the generalizability of the study finding. However, within the limits of the design of the study, the data still provides useful information highlighting the prevalence of developmental dental hard tissue anomalies in the permanent dentition in the study population, and the non-significant association between developmental dental hard tissue anomalies generally and enamel hypoplasia specifically, with caries in the study population.

## Conclusions

Children in Southwestern Nigeria have low clinical prevalence of developmental dental hard tissue anomalies in their permanent dentition. More females and children with middle socioeconomic status had enamel hypoplasia. The presence of developmental dental hard tissue anomalies is significantly associated with poor oral hygiene but not caries in the study population. Children with developmental dental hard tissue anomalies should be treated as children with high risk for poor oral hygiene.

## Abbreviations

APR, adjusted prevalence ratio; DMFT, decayed, filled and missing permanent teeth; ECC, early childhood caries; LGA, Local Government Area; OHI - S, oral hygiene index status; PR, prevalence ratio; WHO, World Health Organization
